# The Hlx homeobox transcription factor is required early in enteric nervous system development

**DOI:** 10.1186/1471-213X-6-33

**Published:** 2006-07-19

**Authors:** Michael D Bates, Dana T Dunagan, Lynn C Welch, Ajay Kaul, Richard P Harvey

**Affiliations:** 1 Division of Gastroenterology, Hepatology and Nutrition, Cincinnati Children's Hospital Medical Center, Department of Pediatrics, University of Cincinnati College of Medicine, Cincinnati, Ohio, USA; 2 Division of Developmental Biology, Cincinnati Children's Hospital Medical Center, Department of Pediatrics, University of Cincinnati College of Medicine, Cincinnati, Ohio, USA; 3 Victor Chang Cardiac Research Institute, St. Vincent's Hospital, Darlinghurst, New South Wales, Australia; 4 University of New South Wales, Kensington, New South Wales, Australia

## Abstract

**Background:**

Development of the enteric nervous system (ENS) requires interactions between migrating neural crest cells and the nascent gastrointestinal tract that are dependent upon genes expressed by both cell compartments. *Hlx*, a homeobox transcription factor gene that is expressed in mouse intestinal and hepatic mesenchyme, is required for normal embryonic growth of intestine and liver, and the *Hlx*^-/- ^genotype is embryonic lethal. We hypothesized that *Hlx *is required for ENS development.

**Results:**

Enteric neurons were identified in *Hlx*^+/+ ^and *Hlx*^-/- ^mouse embryos by immunostaining of embryo sections for the neural markers PGP9.5 and Phox2b, or by staining for β-galactosidase in whole-mount embryos containing the dopamine β-hydroxylase-n*LacZ *transgene. In *Hlx*^+/+ ^embryos, neural crest cells/enteric neurons have moved from the stomach into the intestine by E10.5. By contrast, neural crest cells/enteric neurons remain largely restricted to the lateral stomach mesenchyme of *Hlx*^-/- ^embryos, with only a few scattered neural crest cells/enteric neurons in the intestine between E10.5–16.5.

**Conclusion:**

The Hlx homeobox transcription factor is required for early aspects of ENS development.

## Background

Gastrointestinal (GI) motility requires smooth muscle contraction and relaxation that is regulated in an integrated fashion by the enteric nervous system (ENS), a complex network of ganglia that can function independently of the central nervous system [1]. The ENS is derived embryologically from cells of the vagal and sacral neural crest that migrate into the developing gut via defined pathways, differentiate along a number of lineages (including various neuronal types and glial cells; [2,3]), proliferate [4], and form ganglia [5]. Cells of the vagal neural crest migrate into and along the developing gut proximally to distally, while those of the sacral neural crest colonize the gut from distal to proximal. The differentiation of enteric neurons may continue beyond birth [6,7].

The development and maintenance of the ENS requires cell-cell interactions between the migrating neural crest cells (NCC) and the resident GI epithelial and mesenchymal cells. A number of signaling pathways between mesenchyme and NCC are required for development of the ENS [8-10]. These signaling pathways involve peptides secreted by intestinal mesenchymal cells that act via receptors on NCC. Thus, endothelin-3, glial cell line-derived neurotrophic factor (GDNF), neurturin, neurotrophin-3 (NT-3), and netrin-1, each secreted by intestinal mesenchyme, interact with neuronal endothelin-B, GDNF family receptor (GFR) α-1/Ret, GFRα-2/Ret, p75/TrkC, and deleted in colon carcinoma (DCC) receptors, respectively. However, the extent to which these pathways regulate overlapping or independent populations of enteric nerves is not clear. Targeted inactivation of mouse genes expressing members of the endothelin or GDNF signaling pathways results in ENS malformations as well as other anomalies [11]. Furthermore, mutations of these genes in humans have been associated with ENS malformations such as Hirschsprung disease [8,10,12]. The regulatory mechanisms underlying these signaling pathways remain to be fully elucidated.

Among the important participants in developmental programming are members of the homeobox gene family [13]. The members of this family encode transcription factors that occupy high-level positions in the genetic hierarchy of development, in that the expression of a homeobox gene often initiates a genetic pathway or cascade that regulates cell differentiation and/or proliferation. In intestinal development, many homeobox genes have been shown to be key regulators of a variety of processes. In particular, several homeobox genes expressed by NCC and their cellular progeny have been shown to be important for normal ENS development, including *Phox2b*, *Pax3 *and *Ncx/Hox11L.1 *[14-18], although more recent work suggests that the effects of targeting the latter gene may be due to effects on smooth muscle development [19]. Transcription factors of other families that are expressed in NCC have also been shown to be important for ENS development, such as Sox10 and Mash-1 [18,20-22]. However, no transcription factor genes (of the homeobox family or otherwise) expressed in intestinal mesenchymal cells have to date been shown to be required for ENS development.

*Hlx *is a divergent homeobox transcription factor gene that is highly conserved among mammals, birds, and fish [23,24]. In mouse development, it is expressed most prominently in the mesenchyme of the intestine and liver by E9.5, with peak expression at E10.5–12.5 [25]. A similar pattern of expression is seen in developing chick [23]. Targeted inactivation of the mouse Hlx gene results in an embryonic lethal phenotype in which the intestine and liver are present but do not grow normally [26]; heterozygotes are normal.

We hypothesized that *Hlx *is required for normal development of the ENS, because of its expression in intestinal mesenchyme at a key time for ENS development. To address this hypothesis, we compared the complement of developing enteric neurons in *Hlx*^+/+ ^and *Hlx*^-/- ^embryonic intestinal mesenchyme using specific antisera against neuronal markers and using transgenic markers of developing neurons. We found that the number and distribution of enteric neuronal precursors/neurons is significantly altered in *Hlx*^-/- ^embryos as early as E10.5 as compared to wild-type littermates, demonstrating that the Hlx transcription factor is necessary for early events in ENS development.

## Results

### *Hlx*^-/- ^embryos survive later in gestation on the FVB/N background

**Figure 1:**
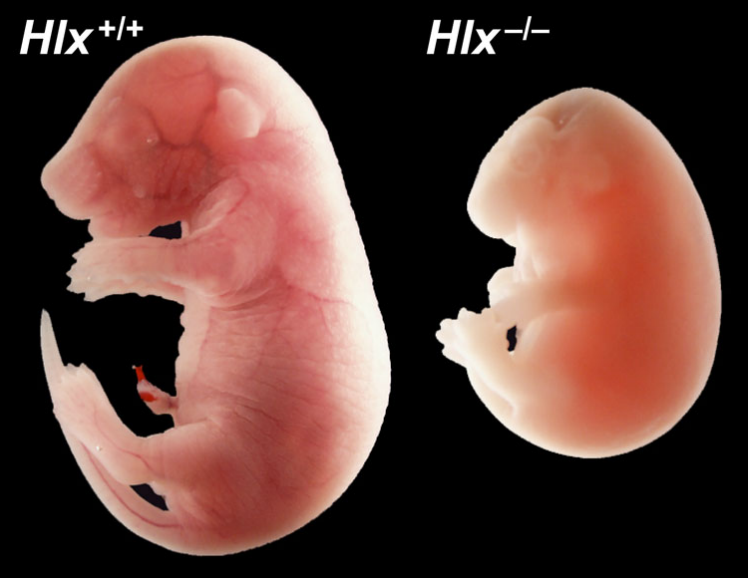
**Comparison of the appearance of littermate *Hlx*^+/+ ^and *Hlx*^-/- ^embryos on the FVB/N background at E17.5**. As compared to wild-type (left), *Hlx*^-/- ^embryos (right) are pale and hydropic. The swelling of the skin on the head makes the head appear abnormal, but internal structures are grossly normal.

On a mixed genetic background (129 × C57BL/6), the *Hlx*^-/- ^genotype is lethal at approximately E15 [26]. To provide a more uniform genetic background for further studies, we backcrossed the knockout allele onto an FVB/N background for ten generations. FVB/N was chosen because mice of this commonly used strain are very fertile (so that large numbers of embryos can be easily obtained), and because it is commonly used to generate transgenic mice. The gross phenotype of *Hlx*^-/- ^embryos before E15 is similar on both genetic backgrounds. However, interestingly, we found dead *Hlx*^-/- ^newborn pups on the FVB/N background. Analysis of embryo genotypes demonstrates no discernible loss of *Hlx*^-/- ^embryos through E18.5 (data not shown). Late-gestation *Hlx*^-/- ^embryos are small and pale compared to their littermates, and they have a hydropic appearance, with the skin ballooned by subcutaneous fluid (Fig. 1), similar to some human newborn infants born with hydrops. This fluid could be explained by the lack of hepatic development resulting in decreased serum concentrations of albumin and other proteins normally synthesized by the liver that contribute to normal intravascular oncotic pressure. The survival of FVB/N *Hlx*^-/- ^mouse embryos until later in gestation suggests that modifier genes play a role in survival in the absence of *Hlx*. This later survival allows a more complete analysis of the consequences of *Hlx *mutation on intestinal development.

### Ontogeny of enteric nervous system development in *Hlx *knockout embryos

**Figure 2:**
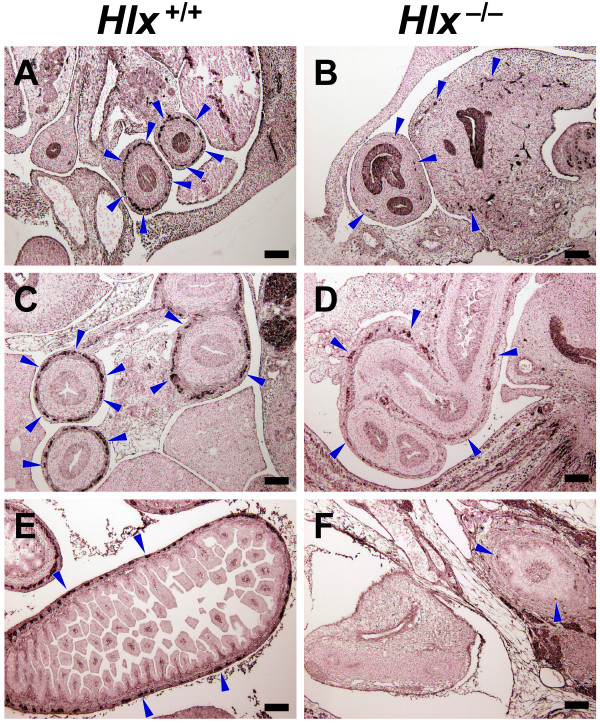
**Ontogeny of ENS development in *Hlx *wild-type and mutant embryos**. Sections from littermate *Hlx*^+/+ ^(panels A, C, and E) and *Hlx*^-/- ^(panels B, D, and F) embryos at E12.5 (panels A and B), E14.5 (panels C and D), and E16.5 (panels E and F) were stained for PGP9.5 as described in *Methods *with the primary antibody at a titer of 1:800. Sections were chosen to identify complementary regions of the GI tract. Examples of PGP9.5^+ ^cells are indicated by blue arrowheads. Black bars, 100 μm. By E12.5, wild-type intestine is well populated with PGP9.5^+ ^cells. Clustering of PGP9.5^+ ^cells is evident thereafter. However, in *Hlx*^-/- ^embryos, only a few PGP9.5^+ ^cells are seen at any timepoint.

Because intestinal mesenchyme is an important regulator of neural crest cell migration, differentiation, and proliferation [5,11], we hypothesized that expression of *Hlx *is required for the normal development of the ENS. To test this hypothesis, we first compared the complement of enteric neurons in *Hlx*^+/+ ^and *Hlx*^-/- ^mouse embryos at E12.5–16.5 by immunohistochemistry using an antiserum against the neuronal marker PGP9.5 [27]. PGP9.5, which has been used as a marker for aganglionosis [28], is a neuron-specific ubiquitin hydrolase that is expressed by enteric neurons. Fig. 2 shows PGP9.5 immunostaining of sections from *Hlx*^+/+ ^and *Hlx*^-/- ^littermate embryos at E12.5–16.5. In wild-type embryos, a complete ring of PGP9.5^+ ^enteric neurons is observed by E12.5 in the intestinal mesenchyme (Fig. 2A). Similar results were observed at E12.5 when staining for peripherin (data not shown). Later, these PGP9.5^+ ^cells coalesce as the ENS matures and ganglia begin to form (Fig. 2C,E). By contrast, only sporadic PGP9.5^+ ^cells are observed in the intestinal mesenchyme of *Hlx*^-/- ^embryos from E12.5–16.5 (Fig. 2B,D,F). This finding is all the more striking when one considers that the *Hlx*^-/- ^embryonic intestine is extremely short compared to wild-type [26], meaning that the PGP9.5^+ ^cells neurons that are present are concentrated in a much shorter length of intestine.

### Role of the Hlx transcription factor in early ENS development

**Figure 3:**
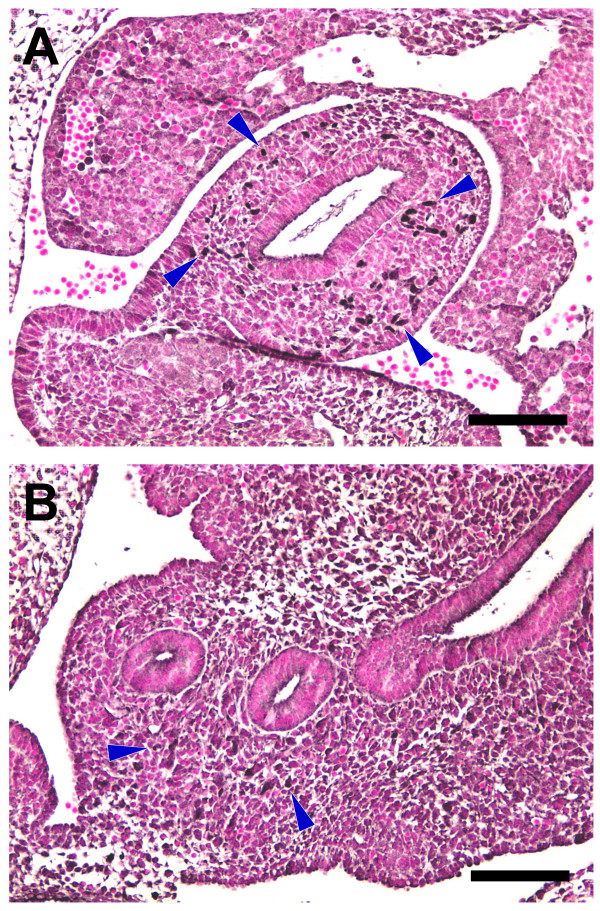
**Lack of Phox2b^+ ^cells in the intestinalmesenchyme of E10.5 *Hlx *knockout embryos**. The figure shows Phox2b staining of sections from littermate *Hlx*^+/+ ^(panel A) and *Hlx*^-/- ^(panel B) embryos at E10.5. Staining was performed as described in *Methods *with the primary antibody at a titer of 1:500. Examples of Phox2b^+ ^cells are indicated by blue arrowheads. Black bars, 100 μm.

**Figure 4:**
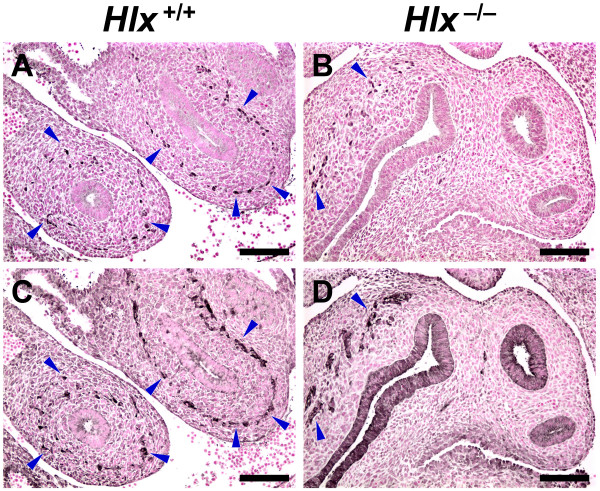
**Phox2b^+ ^and PGP9.5^+ ^cells in E11.5 GI mesenchyme**. Figure shows Phox2b (panels A and B) and PGP9.5 (panels C and D) staining of adjacent histological sections obtained from littermate *Hlx*^+/+ ^(panels A and C) and *Hlx*^-/- ^(panels B and D) embryos at E11.5. Staining was performed as described in *Methods *with the primary antibody at these titers: Phox2b, 1:500; PGP9.5, 1:1,200. Examples of Phox2b^+ ^and PGP9.5^+ ^cells are indicated by blue arrowheads. Black bars, 100 μm.

The observation of an altered PGP9.5^+ ^cell population in *Hlx*^-/- ^embryo suggests that the mutation of *Hlx *results in a lack of NCC/early enteric neurons in the intestine or in an alteration in their differentiation. To explore these possibilities, we compared *Hlx*^+/+ ^and *Hlx*^-/- ^embryos at E10.5–11.5, when ENS development is in its early stages, using antisera against both PGP9.5 and Phox2b [29], a homeobox transcription factor that is expressed by migrating NCC/neural precursors and differentiated enteric neurons. At E10.5, Phox2b^+ ^cells are present, scattered throughout the intestinal mesenchyme in *Hlx*^+/+ ^embryos (Fig. 3A). In *Hlx*^-/- ^embryos, only a few lightly-stained cells are seen in the intestinal mesenchyme (Fig. 3B). One day later, Phox2b^+ ^cells have begun to organize in proximal intestinal loops of *Hlx*^+/+ ^embryos, forming an incomplete ring in the intestinal mesenchymal wall, with the Phox2b^+ ^cells still scattered more distally (Fig. 4A). The distribution of PGP9.5^+ ^cells is the same as that for Phox2b^+ ^cells (Fig. 4C). By contrast, in *Hlx*^-/- ^embryos, Phox2b^+ ^and PGP9.5^+ ^cells are observed only in the mesenchyme of the lateral wall of the stomach and not more distally in the GI tract (Fig. 4B,D).

### Position of enteric neurons along the anterior-posterior axis of the embryonic GI tract

**Figure 5:**
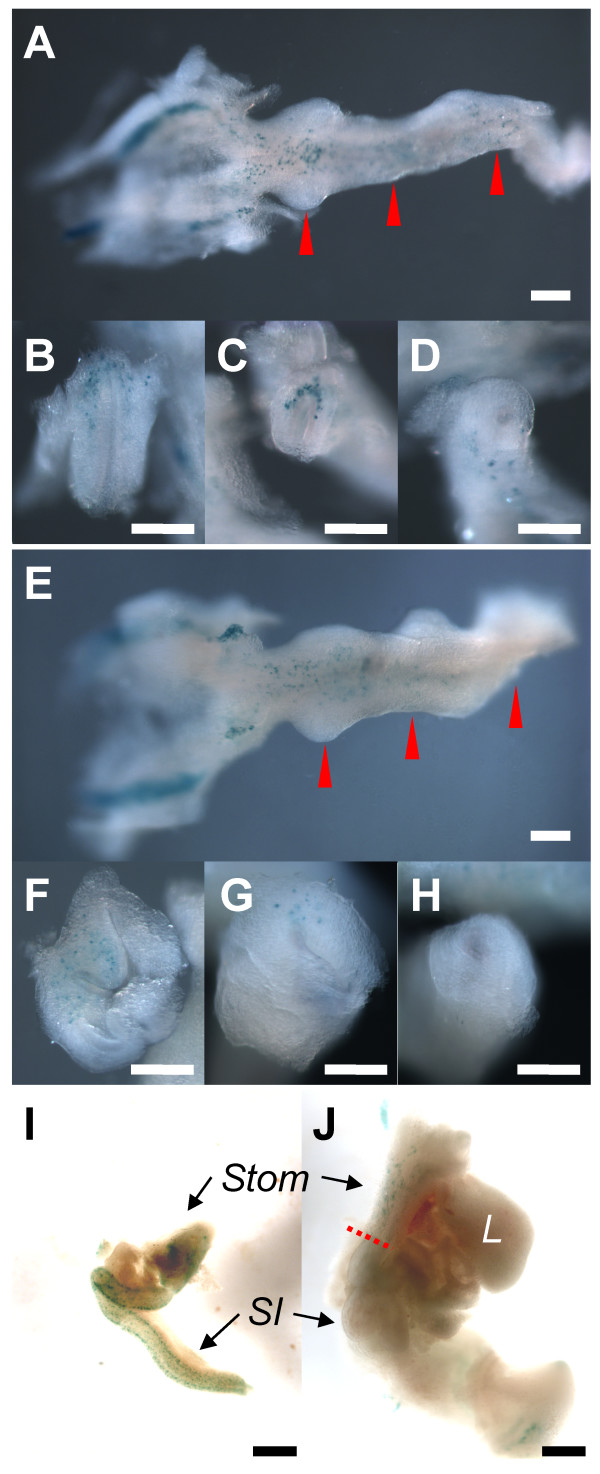
**β-gal^+ ^cells in E10.5 and E12.5 GI mesenchyme**. Littermate DβH-n*LacZ*^+ ^whole-mount embryos were stained for β-galactosidase and GI tissues were dissected out and photographed as described in *Methods*. Panels A-H show dissected GI tracts (panels A and E; left = anterior, right = posterior) and end-on views of the cross-sections (panels B-D, F-H) at the indicated places (red arrowheads). The leftmost cross-sections (panels B and F) are at the level of the nascent stomach. In the DβH-n*LacZ*^+ ^Hlx^+/+ ^(not shown) or *Hlx*^+/- ^embryos (panels A-D), blue β-gal^+ ^cells are present along the length of the GI mesenchyme within the ventral wall. However, in the DβH-n*LacZ*^+ ^*Hlx*^-/- ^embryos (panels E-H), β-gal^+ ^cells are present anteriorly but not posteriorly. White bars, 200 μm. Panels I and J show β-gal^+ ^cells in E12.5 GI mesenchyme. The locations of the stomach (Stom), small intestine (SI), and liver (L) in the preparations are indicated. In the DβH-n*LacZ*^+ ^*Hlx*^+/+ ^embryos (panel I), blue β-gal^+ ^cells are evenly distributed within and along the length of the gut mesenchyme. However, in the DβH-n*LacZ*^+ ^*Hlx*^-/- ^embryos (panel J), there is a concentration of β-gal^+ ^cells in the lateral wall of the gastric mesenchyme, and only scattered β-gal^+ ^cells are seen more distally in the GI tract (beyond the dotted red bar). Black bars, 500 μm.

To identify the position of enteric neurons along the anterior-posterior axis of the developing GI tract more easily, we compared wild-type and *Hlx *knockout embryos that also possess the dopamine β-hydroxylase (DβH)-n*LacZ *transgene [30] in whole-mount preparations. This transgene is expressed in the nuclei of neurons of a variety of lineages, including enteric neurons [30,31]. Fig. 5 compares the position of β-galactosidase-positive (β-gal^+^) cells along the anterior-posterior axis of wild-type and *Hlx *knockout embryos. At E10.5, β-gal^+ ^cells are observed along the small intestine of *Hlx*^+/- ^embryos (Fig. 5A–D). Interestingly, these β-gal^+ ^cells are most prominent in the ventral wall of the stomach and small intestine. Similar results are seen for both *Hlx*^+/+ ^and *Hlx*^+/- ^embryos. However, in *Hlx*^-/- ^embryos, β-gal^+ ^cells are present in the stomach but very few are observed more distally in the small intestine. As in wild-type, β-gal^+ ^cells are in the ventral wall of the GI tract in *Hlx*^-/- ^embryos (Fig. 5E–H). By E12.5, β-gal^+ ^cells are distributed evenly within the intestinal mesenchyme and along the length of the small intestine of *Hlx*^+/+ ^embryos (Fig. 5I). By contrast, β-gal^+ ^cells are largely confined to the gastric mesenchyme, and only scattered β-gal^+ ^cells are seen more distally in the *Hlx*^-/- ^GI tract (Fig. 5J). Interestingly, the domain of β-gal^+ ^cells (red dotted line in Fig. 5J) ends at the anterior boundary of Hlx expression, at the gastric-duodenal border [25].

## Discussion

*Hlx *is a homeobox gene that is required for normal development of the digestive system. As previously described [26], the *Hlx*^-/- ^genotype is embryonic lethal, with embryos exhibiting a short intestine and a very small liver. *Hlx *is expressed in intestinal and hepatic mesenchyme beginning as early as E9.5 [25], where it likely regulates the expression of participants in mesenchymal-epithelial interactions important for growth of the intestine and liver in development. We hypothesized that *Hlx *might also be required for another process in intestinal development in which cell-cell interactions of mesenchymal cell with other elements play a prominent role, namely, development of the ENS. Indeed, we found that *Hlx *is required for early steps in ENS development.

During the course of ENS development, NCC/enteric neurons express various marker proteins in overlapping and dynamic patterns. In addition, the ontogeny of these markers may vary among species. Thus, a variety of markers have been used to study ENS development, and various schemes have been developed to put these data together [32,33]. Markers that have been used for immunological assays include: peripherin, which identifies committed and differentiated peripheral neurons [34]; PGP9.5, which is present in enteric neurons both during and after migration [27]; Phox2b, a homeobox transcription factor that is present in the nuclei of migrating NCC/neural precursors and differentiated neurons [29,33]; nestin, which identifies proliferating neuronal precursors [32]; and tyrosine hydroxylase, which defines the early subpopulation of *Mash-1*-dependent neurons that are transiently catecholaminergic [2,5].

A number of aspects of GI development occur directionally along the anterior-posterior axis of the GI tract, including differentiation of the epithelium and mesenchyme, the organization of these layers into villi, crypts, glands, and smooth muscle, and the development of the ENS. One difficulty with the use of histological sections to study GI development is that findings are difficult to demonstrate in single or even a few sections. This difficulty can be ameliorated by the detection of enteric neurons in whole embryos using transgenic markers or by whole-mount immunohistochemistry [31]. A number of reporter transgenes (expressing β-galactosidase, green fluorescent protein, or the Cre recombinase) have been shown to be expressed in enteric neurons (for review, see [9]), including the DβH-n*LacZ *transgene [30] used in this study. This transgene is expressed in the nuclei of enteric neurons in adult mice [35], and the use of these transgenic mice in developmental studies has also been described [30,36]. They have been used, for example, to demonstrate the defect in neural crest cell migration in *ls*/*ls *mice, which have a spontaneous mutation of the gene encoding endothelin-3 [36].

Our use of complementary approaches, namely, immunohistochemistry in tissue sections and detection of a transgene marker in whole-mount embryos, provides complementary information based on the advantages of each. Results using both methods demonstrate that there is a defect in ENS development in the absence of *Hlx*, with loss of PGP9.5^+^, Phox2b^+^, and DβH-n*LacZ*^+ ^cells within the intestinal mesenchyme. This defect can be detected as early as E10.5, when NCC have just begun moving into the GI tract. PGP9.5^+^, Phox2b^+^, and DβH-n*LacZ*^+ ^cells are largely restricted to the lateral stomach mesenchyme of *Hlx*^-/- ^embryos. Only a few scattered NCC/enteric neurons are observed in the small intestine of *Hlx*^-/- ^embryos between E10.5–16.5, despite being concentrated in the much shorter length of *Hlx*^-/- ^intestine. Because *Hlx *is not expressed in NCC, the defect in Hlx knockout embryos is not neural crest cell-autonomous but instead must alter function of developing intestinal mesenchyme.

**Figure 6:**
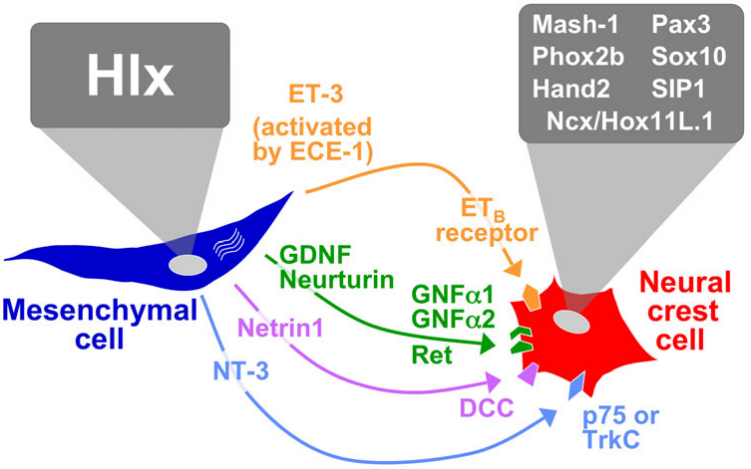
**Transcription factors and signaling pathways regulating enteric nervous system development**. Schematic shows proteins that are known to be required for ENS development. These include transcription factors, ligands, and receptors that are expressed in intestinal mesenchymal cells or NCC/enteric neurons. See text for details.

The ENS forms by migration of NCC into the developing gut. After moving into the gut, these neuronal precursors normally proliferate, differentiate, and organize into ganglia. Work by many groups has identified a large number of genes expressed in NCC/enteric neurons that are required for these processes (Fig. 6). We have found that a transcription factor gene expressed in intestinal mesenchyme, *Hlx*, is required for ENS development in mouse, demonstrating that gene regulation in this cell population is critical for neural crest cell/enteric neuronal processes. Previously, Wolgemuth *et al*. showed that mice overexpressing *HoxA4 *in intestinal mesenchyme develop megacolon [37], although no intestinal phenotype is observed in *HoxA4*-mutant mice [38]. It is likely that the Hlx transcription factor regulates ENS development through transcriptional regulation of one or more mesenchymally-expressed genes that direct these processes. The early defect in ENS development observed in *Hlx*^-/- ^embryos, and the restriction of neurons to the stomach, which does not express *Hlx *[25], implies that the defect most likely results from altered migration of NCC into the intestine. Formally, such altered neuronal migration in *Hlx*^-/- ^intestine could result from the lack of an attractant signal or the presence of a repellent signal. We have been unable to determine a difference in expression of several participants in mesenchymal-neuronal or epithelial-neuronal signaling, including GDNF, endothelin-3, endothelin converting enzyme-1, and Sonic hedgehog, between wild-type and *Hlx*^-/- ^embryos (data not shown). Thus, ENS development may be regulated by Hlx through a heretofore unrecognized signaling mechanism.

## Conclusion

We found that the number and distribution of enteric neuronal precursors/neurons is significantly altered in *Hlx*^-/- ^embryos as early as E10.5 as compared to wild-type littermates. Thus, the Hlx transcription factor is necessary for early events in ENS development. It is likely that Hlx regulates ENS development through transcriptional regulation of one or more mesenchymally-expressed genes that direct these processes.

## Methods

### Mice and embryos

*Hlx *knockout mice [26] that were backcrossed with FVB/N mice (Jackson Laboratories, Bar Harbor, Maine) for at least ten generations were used in this study. For indicated studies, mice also possessing the dopamine β-hydroxylase (DβH)-n*LacZ *transgene [30] were used; founders on a C57BL/6 background, kindly provided by Dr. Raj Kapur (University of Washington), were bred with *Hlx*^+/- ^mice. All studies were approved by the Institutional Animal Care and Use Committee of Cincinnati Children's Hospital Medical Center.

Mouse embryos were obtained by timed overnight matings of *Hlx*^+/- ^mice (with or without the DβH-n*LacZ *transgene), with the morning of the vaginal plug taken to be embryonic day 0.5 (E0.5). Embryos were obtained at the indicated times from pregnant females that were sacrificed by carbon dioxide inhalation (consistent with guidelines of the American Veterinary Medical Association Panel on Euthanasia [39]). Embryos were dissected out in ice-cold phosphate-buffered saline (PBS) and fixed in 4% paraformaldehyde in PBS at 4°C (overnight for embryos for tissue sections, one hour or less for whole-mount β-galactosidase assays). For all experiments, we compared littermate embryos.

Genomic DNA was obtained by proteinase K digestion of tail clippings (weanlings) or yolk sacs (embryos) for PCR detection of the wild-type and *Hlx *knockout alleles [26] and for the *LacZ *gene (primers: forward, 5'-GCATCGAGCTGGGTAATAAGGGTTGGCAAT-3'; reverse, 5'-GACACCAGACCAACTGGTAATGGTAGCGAC-3').

### Immunohistochemistry

The following primary antibodies were used in this study: rabbit anti-PGP9.5 (Accurate Chemical and Scientific Corp., Westbury, NY) at dilutions of 1:800–1:1200; and rabbit anti-Phox2b [29] (kind gift of Dr. Jean-François Brunet, CNRS, École Normale Supérieure, Paris) at a dilution of 1:500.

Paraformaldehyde-fixed littermate *Hlx*^+/+ ^and *Hlx*^-/- ^embryos were paraffin-embedded, and 4 μm sections were placed on positively charged slides. After deparaffinization, removal of endogenous peroxidase, and antigen retrieval, tissue sections were assayed as follows. For identification of PGP9.5-positive or Phox2b-positive cells, sections were incubated for 45 min at room temperature with primary antibody. Adjacent sections with primary antibody omitted were processed as negative controls. The Dako LSAB 2 System (DakoCytomation, Carpinteria, CA) was used to detect the antigen-antibody complexes. Sections were incubated at room temperature with biotinylated secondary antibody for 30 min, followed by streptavidin-horseradish peroxidase for 30 min. Peroxidase was detected using 3,3'-diaminobenzidine, and the enzymatic reaction product was enhanced with nickel cobalt to yield a black precipitate. Sections were then counterstained with nuclear fast red for 2 min.

We also attempted to detect nestin expression in E11.5 intestine using mouse anti-nestin (BD Biosciences PharMingen, San Diego, CA), at a dilution of 1:200, with the M.O.M. Peroxidase kit (Vector Labs, Burlingame, CA). For both *Hlx*^+/+ ^and *Hlx*^-/- ^embryos, staining was observed in the neural tube and dorsal root ganglia, but no intestinal staining was observed.

### Detection of β-galactosidase in transgenic embryos

Whole-mount embryos were fixed with 4% paraformaldehyde in PBS for 15–60 minutes and stained by the method of Sanes *et al*. [40,41]. Thus, fixed embryos were incubated in stain solution [0.9 mg/ml 5-bromo-4-chloro-3-indolyl-β-D-galactopyranoside (X-gal), 7.5 mg/ml potassium ferricyanide, 8.6 mg/ml potassium ferrocyanide, 3.5 mg/ml spermidine, 2 mM MgCl_2_, 0.01% sodium deoxycholate, 0.017% NP-40 in PBS] at 37°C until color was sufficiently developed (typically 4 hours-overnight). Embryos were then washed with ice-cold PBS and stored at 4°C until dissected within 1–2 days.

### Photomicroscopy

Digital photomicrographs of sections and whole-mount embryos or dissected GI tracts were obtained using an Olympus BX41 stage microscope or an Olympus SZX12 zoom stereo microscope, respectively, using a SPOT Insight Color digital camera (Diagnostic Instruments, Sterling Heights, MI) and SPOT imaging software for Macintosh.

## Authors' contributions

MDB directed the study, analyzed the data, and drafted the manuscript; DTD performed immunohistochemical experiments and embryo staining and assisted with animal breeding; LCW performed immunohistochemical experiments and embryo staining and assisted with animal breeding; AK assisted in the design of the study and performed immunohistochemical experiments; and RPH provided knockout mice and expertise regarding their phenotype. All authors read and approved the final manuscript.
